# Inhibition of IL-17 ameliorates keratinocyte-borne cytokine responses in an in vitro model for house-dust-mite triggered atopic dermatitis

**DOI:** 10.1038/s41598-023-42595-z

**Published:** 2023-10-03

**Authors:** Juliane Haertlé, Petra Kienlin, Gabriele Begemann, Thomas Werfel, Lennart M. Roesner

**Affiliations:** 1 Department of Dermatology and Allergy, Hannover Medical School (MHH), Carl-Neuberg-Str.1, 30625 Hannover, Germany; 2 Cluster of Excellence RESIST (EXC 2155), Hannover Medical School (MHH), Hannover, Germany

**Keywords:** Skin diseases, Cytokines

## Abstract

A subgroup of patients suffering from atopic dermatitis (AD) does not respond to biologics therapy targeting the key players of type-2 inflammation, and it is an ongoing discussion whether skin-infiltrating Th17 cells may underlie this phenomenon. This study aimed to investigate the potential of allergen-induced, immune-cell derived IL-17 on the induction of inflammatory processes in keratinocytes. Peripheral blood mononuclear cells derived from respectively sensitized AD patients were stimulated with house dust mite (HDM) extract and cell culture supernatants were applied subsequently in absence or presence of secukinumab to primary human keratinocytes. Hereby we confirm that the immune response of sensitized AD patients to HDM contains aside from type-2 cytokines significant amounts of IL-17. Blocking IL-17 efficiently reduced the stimulation-induced changes in keratinocyte gene expression. IL-17-dependent transcriptional changes included increased expression of the cytokines IL-20 and IL-24 as well as Suppressor of Cytokine Siganling 3 (SOCS3), a negative feedback-regulator of the STAT3/IL-17/IL-24 immune response. We conclude that the immune response to HDM can induce pro-inflammatory cytokines from keratinocytes in AD, which in part is mediated via IL-17. Targeting IL-17 may turn out to be a reasonable alternative therapy in a subgroup of patients with moderate to severe AD and HDM sensitization.

## Introduction

Atopic dermatitis (AD) represents one of the most common inflammatory, relapsing–remitting and pruritic skin disorder affecting 2–4% adults in Germany and up to 10% worldwide^1–3^. Despite receiving various systemic therapies, patients still have unmet therapeutic needs in some cases^4,5^. This makes long term management challenging resulting in adverse effects on qualitiy of life^6,7^. Furthermore, AD appears to be multifactorial at the pathophysiological level including genetic factors, immunological predispositions, and non-genetic barrier mechanisms (environmental factors, toxic, phototoxic), resulting in epidermal dysfunction and a type-2 biased immune response. In the last years various efforts have been made to understand the individual subcellular signalling-pathways involved in the inflamatory response and the ability to target specific key molecules^8–10^. Immunologically, two subtypes of AD can be distinguished: the extrinsic and the intrinsic type. In the extrinsic type, elevated IgE levels and sensitisation to food and/or environmental allergens are found^11^. In addition, regarding endotypes, it has been proposed to define subgroups with regard to serum mediators (e.g. IL-1, IL-4, IL-13, thymic stromal lymphopoietin, IFN-α, tissue inhibitor of metalloproteinases 1, vascular endothelial growth factor, IFN-β, IL-1, and further epithelial cytokines), total IgE levels as well as allergen-specific IgE levels^8^. Consequently, it will become increasingly important in the future to identify patient populations, in terms of AD endotypes, that can benefit from targeted therapies^8,12^.

In addition to skin-infiltrating Th2-polarized T cells as a key factor for allergic inflammation it is meanwhile accpeted that interleukin 17 (IL-17), produced mainly by Th17 cells, plays a role in the T-cellular response to allergens^12,13^. IL-17 coordinates inflammation in local tissues by induction of pro-inflammatory cytokines and chemokines in keratinocytes^14,15^. AD is considered a polar Th2 disease in the acute phase, with a partial shift to Th1 during the chronic phase. Blocking the Th2 response with dupilumab by targeting the IL-4/IL-13 signaling pathway could result in a shift towards a Th1/Th17 phenotype, leading to an inflammatory cytokine cascade and eventually psoriatic skin plaques^16^.

Increased numbers of Th17 cells in skin biopsies of acute skin lesions and PBMCs (Peripheral Blood Mononuclear Cells) from patients with AD have been described. Furthermore, the amount of Th17 cells was also associated with the severity of AD^17,18^.

In a subgroup of patients suffering from AD, high-titered specific IgE and T-cell immune responses against house dust mites (HDM, e.g. *Dermatophagoides* spp.) allergens are detectable^19^. In previous studies we described T cells specific to two of the major allergens of *D. pteryssinus* to be of a Th2, Th17 and Th2/Th17 phenotype in sensitized AD patients^20^. Based on this, antagonizing IL-17 might have beneficial effects particulary in patients with AD and specific IgE againts HDM allergens^10^. IL-17 is established as a target molecule to reduce inflammation in psoriasis. Here therapy with IL-17 inhibitors such as Secukinumab, a fully human IgG1κ antibody that specifically binds to IL-17A (and IL-17A/F) was introduced into clinical routine in the year 2015.

Systemic therapy with biologics started for AD in September 2017 when the IL-4R blocking antibody dupilumab was approved^21,22^, and continued 2021 with the approval of the anti-IL-13 antibody tralokinumab, both targeting the type-2 inflammation axis. Currently, there are many other biologics and innovative small molecules in phase II and phase III clinical trials shown a promising progess in the treatment of AD^4,23^.

Many but not all AD patients benefit from type-2 inflammation-targeted therapy, and it is a clear need to define commonalities of dupilumab non-responders to tackle this issue. The theory, that an IL-17-inflammation bias could account for this in a subgroup of patients is underlined by the raising numbers of case reports describing emergence of IL-17-driven diseases under dupilumab therapy^24–26^. In a recent review article, 31 ‘case series’ and ‘case reports’ of dupilumab-associated psoriasis and psoriasiform manifestations were published between January 2018 and September 22^27^.

The underlying pathophysiological mechanisms that could explain the occurrence ot psoriais following dupilumab treatment remain elusive^28^. Additionally, recent experimental studies have shown that IL-4 can act directly on T cells, dendritic cells and keratinocytes^29–31^. Two clinical trials investigated a potential benefit of IL-17 blockade by secukinumab treatment in AD patients, registered under NCT03568136 and NCT02594098. Latter trial has published results recently and conclude that a targeted solo-blockade of IL-17 does not lead to bettering of AD clinical symptoms or laboratory parameters^32^. However, the authors suggest that a combinatory therapy targeting type-2 inflammation in parallel to IL-17 may nevertheless be beneficial. A mouse model of allergic asthma have also been reported to benefit from therapy targeting IL-13 and IL-17 in parallel^33^.

This study aimed to model the skin inflammation in AD in which allergen-specific T cells infiltrate the skin and elicit pro-inflammatory mediators, leading to immune activation of keratinocytes. It is of special interest to investigate whether an IL-17-targeted therapy can effectively suppress the pro-inflammatory influence from T-lymphocytes on keratinocytes of the skin in order do be able to treat patients with specific sensitisations against HDM allergens even more effectively in the future by means of a targeted (add-on) therapy.

## Results

### PBMC of sensitized donors with AD respond with IL-17 to house dust mite stimulation

**Figure 1:**
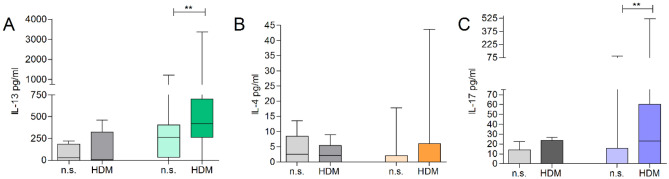
Short term T cell lines of sensitized AD patients respond with type-2 cytokines as well as IL-17 to HDM stimulation. PBMC derived from patients suffering from AD (n = 30) as well as from non-atopic control donors (grey, n = 8) were stimulated in vitro with HDM extract (10 µg/ml) or left untreated (not stimulated, n.s.). After 5 days, levels of secreted IL-4, IL-13, and IL-17 were determined within the cell culture supernatants. **p < 0.01.

The cellular immune response to HDM in respectively sensitized individuals has been shown to comprise, aside from type-2 cytokines, also IL-17. To investigate, if this effect can be monitored in short term T cell lines, PBMC from 30 AD patients with HDM sensitization as well as 8 non-atopic control donors were isolated and short-term T cell lines were generated by in vitro stimulation with HDM extract. After 5 days, secretion of the cytokines IL-4, IL-13 and IL-17 was quantified by ELISA. An upregulation of IL-13 as well as IL-17 was detected in patients with AD and HDM sensitization after antigen stimulation. In non-atopic controls no cytokine secretion could be induced by antigen stimulation (Fig. 1).

### Identification of target immune mediators of IL-17 in primary human keratinocytes

**Figure 2:**
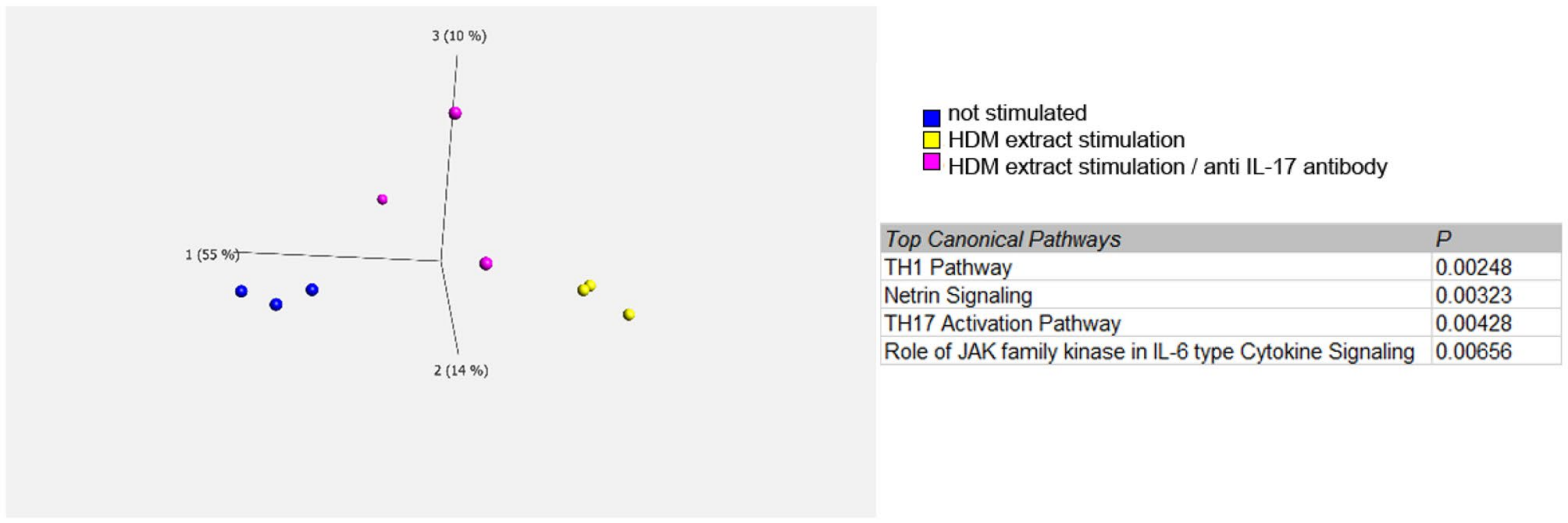
Principal component analysis of transcriptomes of keratinocytes cultured in cell culture supernatants derived from unstimulated or HDM-stimulated immune cells in presence or absence of blocking anti-IL17-antibodies. Principal component analysis (PCA) was performed and canonical pathways were built on data filtered by a two-group-comparison HDM-extract stimulation vs not-stimulated cells, *p* < *0.05*. αIL-17 was added relative to rhIL-17, 500x.

In order to investigate the effect of HDM-induced, T-cell borne IL-17 on keratinocytes, we analyzed the transcriptome of keratinocytes responding to this stimulus. Therefore, three independent patient-derived and HDM-stimulated PBMC cell culture supernatants with a distinct content of HDM-induced IL-17 (compare Supplemental Fig. 2) were applied to primary human keratinocytes in presence or absence of secukinumab. The concentration of the antibody was adapted to the amount of IL-17 present in the respective cell culture supernatant and determined upfront (Supplemental Fig. 3). Keratinocytes cultured in control medium served as control (not stimulated, n.s.). Principal component analysis of transcriptomes showed strong separation of keratinocytes cultured in HDM-stimulated T cell culture supernatants from non-stimulated samples. HDM-stimulation with secukinumab treatment were clustered in between of them, suggesting a distinct effect of HDM-induced, T-cell borne IL-17 on keratinocytes. Canonical pathways specific for this difference included the Th17 activation pathway (Fig. 2).

### IL-17 leads to increased expression of IL-20 and IL-24 in keratinocytes, which can be ameliorated by IL-17A specific antibodies

**Figure 3:**
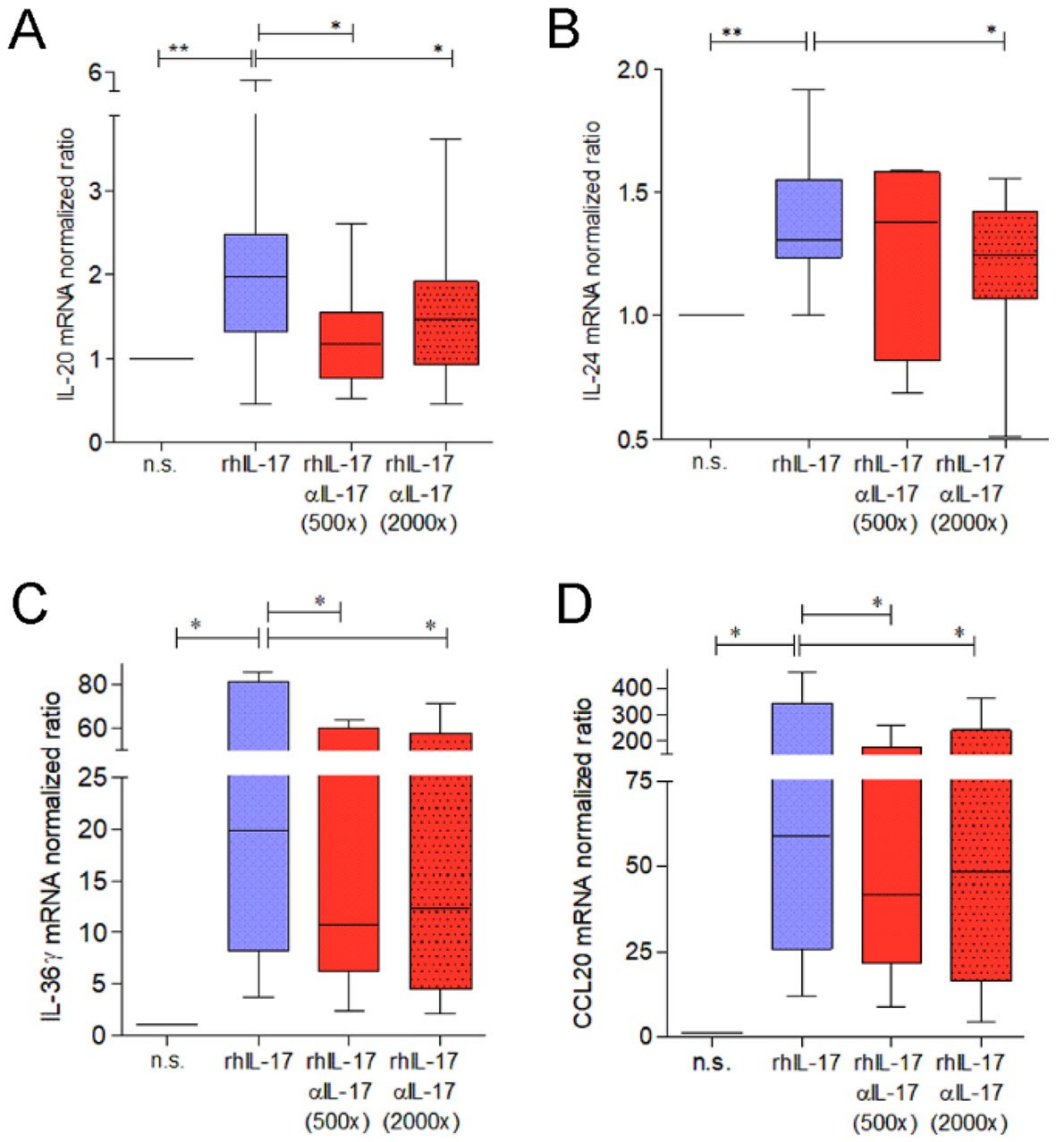
Increased expression of Th17 pathway key mediators upon IL-17 stimulation in keratinocytes. Gene expression of inflammatory mediators as depicted on the y-axes was determined by realtime RT PCR. (**A**): IL-20: n = 14, (**B**): IL-24: n = 10, (**C**): IL-36γ: n = 6, (**D**): CCL20: n = 6. Differences were calculated by Wilcoxon´s rank sum test (**p < 0.01, *p < *0.05*). *rh* recombinant human, *n.s.* non-stimulated. αIL-17 was added relative to rhIL-17, 500 × or 2000 × as indicated.

To further identify key immune mediators of the Th17 pathway in primary human keratinocytes, we aimed next to investigate the expression of several central mediators by realtime RT PCR. The Th17 activation pathway is orchestrated by the Signal Transducer and Activator of Transcription 3 (STAT3). Cytokines of the IL-10 family have been shown to be expressed by keratinocytes and to signal through the STAT3 pathway^34^. Therefore, we decided to investigate the gene expression of the IL-17 target cytokines IL-20 and IL-24, as well as the STAT3-targets SerpinB4 and CCL20, and the cytokines IL-36γ and CXCL8 (IL-8), which have been shown to play roles in STAT3 activation. IL-20 and IL-24 were found to be markedly elevated after stimulation of keratinocytes with rh IL-17, as well as CCL20 and the cytokine IL-36γ (Fig. 3A–D, blue boxes). SerpinB4 showed upregulation in some experiments, as did CXCL8, however, these effects did not reach statistical significance in this set of experiments (data not shown). Pre-incubation of keratinocytes with secukinumab led to significant reductions of IL-20, IL-24, IL-36γ, and CCL20 expression levels (Fig. 3A–D, red boxes).

### HDM-induced, T cell-derived immune mediators lead to increased expression of IL-20 and IL-24 in keratinocytes, which can be ameliorated by IL-17A specific antibodies

The next step was to check whether the increase in the elevated expression levels could also be induced by antigen-stimulated cell culture supernatants of IL-17-producing T cells from patients with AD and HDM sensitization. Since the aim of this project was to investigate a possible positive effect of blocking IL-17, only those donor samples were considered in following experiments in which antigen stimulation led to an induction of IL-17 secretion compared to the non-stimulated sample (n = 26). Secukinumab was applied as before. Culture supernatants from equivalently treated T cells of non-sensitized control donors were used for comparison irrespective of their IL-17 concentration.

**Figure 4:**
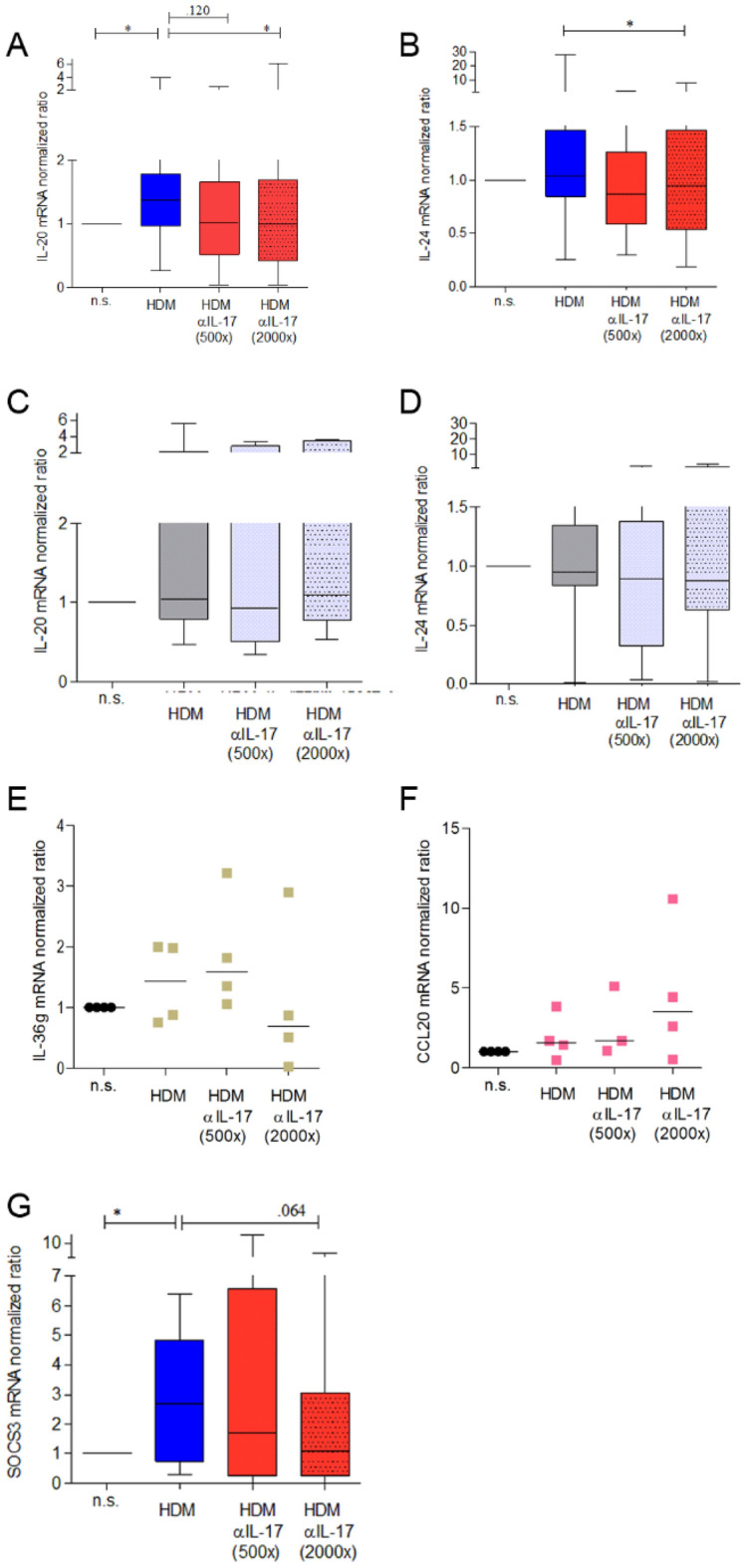
Increased expression of Th17 pathway key mediators in keratinocytes upon cultivation in cell culture supernatants of HDM-stimulated PBMC derived from sensitized AD patients. Primary human keratinocytes were cultivated in cell culture supernatants of HDM-stimulated PBMC (HDM) derived from: (**C**,**D**), non-atopic donors (greyscale) or (**A**,**B**,**E**–**G**) sensitized AD patients for 5 h in presence or absence of secukinumab (500 × or 2000 × compared to IL-17 content of cell culture supernatant). Gene expression of inflammatory mediators as depicted on the y-axes was determined by realtime RT PCR. IL-20: n = 26, IL-24: n = 26, CCL20: n = 4, IL-36γ: n = 4, SOCS3: n = 8. Differences were calculated by Wilcoxon´s rank sum test (*p < *0.05).* *n.s.* non-stimulated.

By this, keratinocytes showed increased mRNA expression of the pro-inflammatory cytokine IL-20 when incubated with patient-derived T cell culture supernatants, and pre-incubation with secukinumab at 2000-fold concentration inhibited this upregulation (Fig. 4A). Gene expression of IL-24 was reduced significantly by secukinumab concentrations 2000-fold compared to IL-17 levels of the respective cell culture supernatant (Fig. 4B). In comparison, HDM stimulated cell culture supernatants derived from healthy control donors had no effect on the expression of the keratinocyte inflammatory mediators IL-20 and IL-24 (Fig. 4C,D). However, no upregulation of gene expression of SerpinB4, CCL20, CXCL8, and IL-36γ was detectable after incubation with patient-derived, HDM-stimulated cell culture supernatants (Fig. 4E,F, data for SerpinB4 and CXCL8 not shown).

Enhancing suppressor of cytokine signaling (SOCS) 3 expression represents an important molecular mechanism to inhibit gp130-dependent STAT3 activation, and it has been shown before to function as a negative feedback to prevent uncontrolled inflammation^35^. In our hands, SOCS3 was upregulated in keratinocytes in response to the incubation with HDM-induced, IL-17 containing T cell culture supernatants, which was ameliorated by secukinumab by trend (*p = 0.064*) (Fig. 4G).

## Discussion

The need to define endotypes of the multifactorial and multi-facetted disease AD has been approached with the classification of the intrinsic vs. the extrinsic and the autoreactive type, the early- vs. late-onset type as well as ethnically different types^8,36^. Intrinsic, autoreactive as well as Asian AD have been proposed to rely to certain extent on IL-17-inflammation^37,38^. Confirming earlier studies, IL-17 expression is detectable in lesional AD skin by immunohistochemistry (see supplemental Fig. 4). Ungar et al. described carefully that a therapy that targets exclusively IL-17 does not lead to clinical bettering of AD. This trial was accompagnied by an intense laboratory program covering immunohistochemical analysis of immune cell infitration into the skin and transcriptomic cellular responses, showing no effect of therapy^32^. Animal models point out that a combination of targeted therapies may be a promising approach in personalized medicine, and case reports have not raised concerns so far.^33,39^.

In vitro studies have shown that focussing on driver T-cell-subsets, such as skin-homing^36^ or antigen-specific T cells^40,41^ bears the potential to decipher pathways that are otherwised covered by the vast amount of other T cell specificities. Here, we tried to model the skin inflammation in AD in which allergen-specific T cells infiltrate the skin^42^ and elicit pro-inflammatory mediators, leading to immune activation of keratinocytes. ^43^ We describe that the HDM allergen-induced pro-inflammatory immune cell-derived milieu leads to the induction of pro-inflammatory mediators from keratinocytes, harbouring the cytokines IL-20 and IL-24.

IL-20 and IL-24 both belong to the family of IL-10 cytokines. Both have been reported to be of importance in the pathogenesis of chronic inflammatory skin diseases and can readily be detected in respective tissue samples^44^. Both cytokines can transmit signals through the IL-20R1/IL-20R2 and the IL-22R1/IL-20R2 heterodimer complexes. Aside from keratinocytes, IL-24 is produced by different immune as well as epithelial cell types, including myeloid cells, mast cells as well as Th2 cells after induction by several stimuli including IL-17A but also the type-2 cytokines IL-4, IL-13, and IL-31^45^. In human keratinocytes, it leads to the activation of STAT3 and thereby drives type-3 inflammation while counteracting in parallel on the intactness of the skin barrier via lowered expression of barrier proteins such as filaggrin^46^. Further on, it was demonstrated that IL-20 and IL-24 are partially responsible for the effects that IL-31 has in deregulating the skin barrier in AD^47^. The importance of IL-20 for the skin is reflected by the observation that overexpression in mice is lethal due to defective skin formation.

Further it was shown in a mouse model of AD that the negative effect of IL-24 on IL-1β expression in keratinocytes may facilitate *Staphylococcus aureus* growth on the skin which is regarded as a hallmark of AD^48^.

IL-10-familiy cytokines also induce a negative feedback loop to control STAT3 activation via SOCS3^49–51^, which is an important pathway of epithelium homeostasis^52^. In line with this, knockout of SOCS3 in keratinocytes led to severe inflammation displaying epidermal hyperplasia, hyperproduction of IgE and high levels of IL-6 and IL-10-family cytokines including IL-20 and IL-24^53^. Therefore it can be assumed, that the increased levels of SOCS3 in our set of experiments represent the negative feedback loop to constrain the exaggerated IL-20/24-STAT3-mediated immune response.

Interestingly, the incubation of cell supernatants of HDM stimulated PBMC with secukinumab did not show an effect on the gene expression of several targets investigated in this study. Since in our model, the HDM-induced immune cell culture supernatant that was applied to stimulate keratinocytes contains a mixture of immune cell mediators, it can be hypothesized that effector molecules aside from IL-17 induce their expression. Our model thus reflects the situation in vivo and may further on explain the lack of effect of a secukinumab mono-therapy in AD clinical trials. More precise, IL-26, another Th17 cell-derived cytokine, has been reported to upregulate expression of CXCL8 (IL-8) as well as CCL20 and could account for the lack of effect of secukinumab in our study^54^. IL-24 can also be upregulated by type-2 cytokines, which could explain the absence of downregulation of IL-20 and IL-24 in NCT02594098. Following this hypothesis, a combinational therapy targeting type-2 and IL-17 inflammation could represent a promising approach to ameliorate skin inflammation.

The production of IL-17 mirrored previous observations, where in vitro stimulation of PBMC with the HDM major allergens Der p 1 and Der p 2 led to an immune response including IL-17. Of note, in those experiments no IL-17 was detectable in equally cultivated cells, which did not proliferate upon HDM allergen stimulation. This suggests a specific role of type-3 inflammation in response to HDM and excludes the possibility of cell culture artifact^20^.

The overweight of IL-13 compared to IL-4 observed here could be due to the fact that primarily adult patients with chronic AD were recruited, and a selective significant up-regulation of IL-13 but not IL-4 mRNA has been reported for subacute and chronic AD^55^. Furthermore IL-4 and IL-13 cooperate in promoting Th2 responses, having both overlapping and additive roles^56^. Thus, stimulated T-cells seem to react more sensitively and faster to IL-4 than to IL-13. However, over time and T cell differentiation, there is a selective upregulation of IL-13 and in parallel a markedly lower expression level of IL-4 observable^57^.

RNA sequencing was performed as part of the study. The canonical signalling pathways included the Th17 activation pathway, the Janus kinase (JAK) signalling pathway and the Th1 signalling pathway. The JAK- STAT pathway plays a central role in modulating AD. In particular, T cellular responses are mediated by JAK-STAT signalling. In the meantime, JAK inhibitors have found their way into the clinical routine of AD therapy and are being used successfully^58^. With regard to the Th1 signalling pathway, it is well known that in the course of the atopic inflammatory reaction, a transition into the chronic phase takes place, in which the Th1 cytokines (IFN- γ, IL-12) are commonly observed.

Taken together, our study shows that the immune cell response to house dust mite in sensitized AD patients is capable of inducing gene expression of pro-inflammatory mediators in keratinocytes, and that this relies to a certain extent on IL-17. Although the overall predominance of the type-2 immune response in AD has been described using Omics techniques^59^ and clinical trials demonstrated that a therapy targeting IL-17 only is not successful^32^, this study describes the pro-inflammatory potential of IL-17 in skin inflammation after allergen-challenge. Regarding the subgroup of patients that does not benefit from type-2 inflammation-directed therapy these findings should be taken into consideration.

## Material and methods

### Patients and sera

Adult patients with AD according to the criteria of Hanifin and Rajka^60^ and relevant levels of specific IgE to HDM (CAP class ≥ 3, ImmunoCAP, Thermo Fisher Scientific, Uppsala, Sweden) were recruited at the Department of Dermatology and Allergy at Hannover Medical School. Patients had a median age of 38.5, mean total serum IgE of 3396 kUl^−1^, a mean disease severity as determined by SCORAD (SCOring Atopic Dermatitis) of 32.2 (indicating moderate AD) and were not under systemic immunosuppressive treatment^21^. Heparinised whole blood samples were taken from AD and non-atopic control subjects. Details are depicted in Supplemental Fig. 1. All patients gave their written informed consent.

### Preparation of cell culture supernatants of antigen-stimulated PBMCs

PBMCs were isolated by density-gradient centrifugation and short-term T cell lines were generated according to an established protocol^20^. Therefore, 2 × 10^6^ PBMC were cultured in presence or absence of HDM extract (10 µg/ml, Citeq Biologics, Groningen, The Netherlands) at a density of 2 × 10^6^/ml in Iscove´s medium (Biochrom KG, Berlin, Germany) supplemented with 4% human heat-inactivated AB serum, 2 mM glutamine, 50 mg/ml of gentamicin, 100 mg/ml penicillin and streptomycin, and nonessential amino acids. The short-term T cell lines were incubated at 37 °C for 5–7 days after stimulation. After incubation the cell lines were centrifuged for 10 min at 3000 × *g* and the supernatants used in subsequent experiments.

### ELISA

ELISAs were performed according to the manufacturers’ instructions and analysed using a plate reader (FluoStar optima, BMG Labtech, Ortenberg, Germany). ELISA kits for the detection of IL-17, and IL-13 were purchased at R&D (Minneapolis, MN, USA) and IL-4 at Thermo Fisher Scientific (Waltham, USA).

### Stimulation of human primary keratinocytes with t-cell supernatants and IL-17A antibody

Primary cultures of normal human keratinocytes were prepared from foreskin resections of anonymous donors at the Department of Immunodermatology and Experimental Allergy Research at Hannover Medical School or purchased (NHEK-Neo, Human Epidermal Keratinocytes, Neonatal, Single Donor, Lonza, Basel, Switzerland)^61^.

For preparation, 5 × 10^4^/ml cells were plated in serum-free keratinocyte growth medium (Keratinocyte Growth Medium 2 Kit; PromoCell GmbH, Heidelberg, Germany) in a 24 well plate. The keratinocytes were incubated at 37 °C for 2 days before recombinant human (rh) IL-17 (5 ng/ml, R&D Systems, Minneapolis, Minnesota, USA) or T-cell culture supernatants were added and incubated for 5 h at 37 °C. Subsequently, the supernatant was aspirated and 400 µl of lysis buffer (guadinium thiocyanate) was added to each well. After 3 min the supernatants were frozen at − 80 °C. T-cell culture supernatants from patients were selected which showed an increased IL-17 content in the cell culture supernatant after antigen stimulation (see Fig. 1C). In a subset of experiments, T-cell culture supernatants were pre-incubated for 1 h at 37 °C with a fully-human IgG1κ anti-IL-17-antibody (Secukinumab, Novartis, Basel, Switzerland). IL-17A antibody was applied in a concentration adapted to the IL-17-quantity in the cell-culture supernatant, in 500-fold and 2000-fold quantities.

### RNA isolation and transcript analysis

Total cellular RNA of keratinocytes was extracted with the InnuPREP Micro RNA Kit (Analytik Jena, Jena, Germany) according to the manufacturer’s instructions.

Selected transcriptomes of keratinocytes were analyzed by RNA sequencing at the Research Core Unit Genomics (RCUG) of Hannover Medical School. Principal component analysis (PCA) was performed on data filtered by a two-group-comparison HDM-extract stimulation vs not-stimulated cells, p < 0.05, calculated by Qlucore Omics Explorer V3.8 (Qlucore, Lund, Sweden). Canonical pathways were built by Ingenuity pathway analysis (Qiagen).

The cDNA was synthesized by reverse transcription with the Quantitect Reverse Transcription Kit (Qiagen, Hilden, Germany). Real-time quantitative LightCycler PCR (Roche Molecular Biochemicals, Mannheim, Germany) was performed with Quantitect primer assay for RPS20 (QT00003290), IL-20 (QT00044905), IL-24 (QT00059059), using SYBR Green according to the manufacturer's instructions (Roche, Basel, Switzerland). The amount of the target mRNA relative to the amount of the reference RPS20 mRNA in the same sample was calculated using the Relative Quantification Software (Roche Molecular Biochemicals).

### Data and statistical analysis

For statistical analysis GraphPadPrism (GraphPad Prism, Version 5.0.0, La Jolla, USA) was applied. Comparing two groups, the Wilcoxon matched pairs test was applied. Results with a p-value < 0.05 were considered statistically significant (*p < 0.05; **p-value < 0.01).

### Ethics declarations

The study was conducted according to the declaration of Helsinki and approved by the Ethics Commitee of Hannover Medical School (Nr. 8303_BO_S_2019).

### Supplementary Information

Supplementary Figures.

## Data Availability

The datasets generated during and/or analysed during the current study are available from the corresponding author on reasonable request.
