# Supplementary material for: Inhibition of IL-17 ameliorates keratinocyte-borne cytokine responses in an in vitro model for house-dust-mite triggered atopic dermatitis
Source: Sci Rep. 2023 Oct 3;13:16628. doi: 10.1038/s41598-023-42595-z (PMC10547677; doi:10.1038/s41598-023-42595-z)
Supplement: Supplementary file 1 — Supplementary Figures. [file 41598_2023_42595_MOESM1_ESM.docx]

**Supplemental Figures**


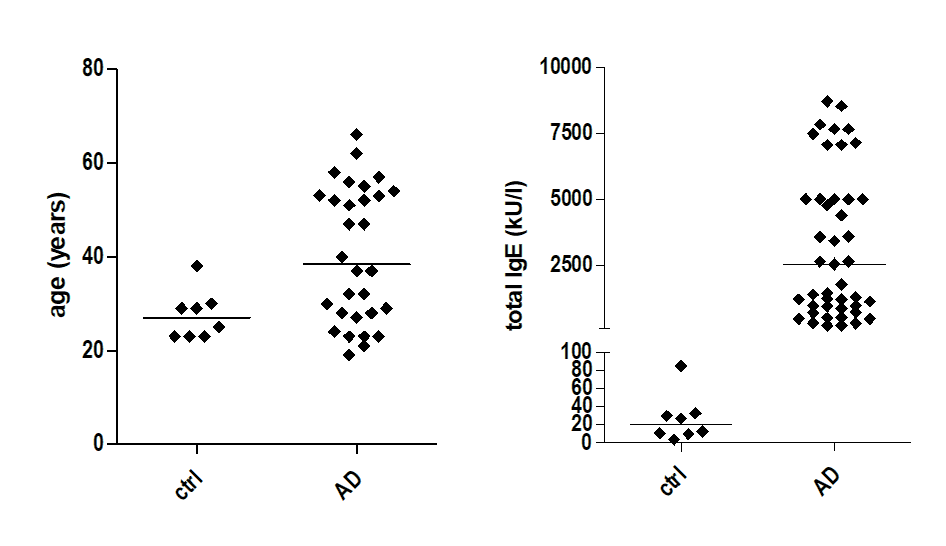


**Supplemental Figure 1. Characteristics of the patients involved.** Both cohorts showed a balanced gender distribution.Age (years), female gender (%), as well as total IgE (kU/l) are depicted. ctrl: healthy, non-atopic donors, n=8; AD: atopic dermatitis, n=30


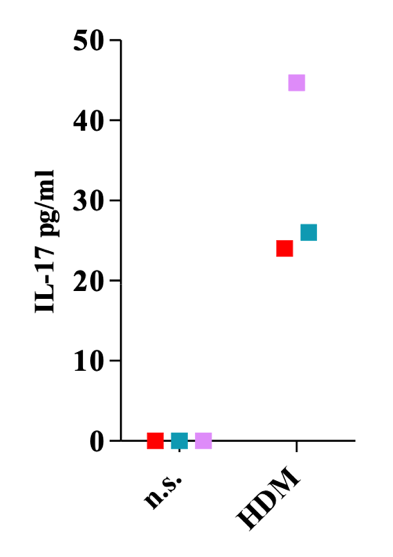


**Supplemental Figure 2. Level of IL-17 in three exemplary patient-derived short-term T cell line cell culture supernatants.** IL-17 levels with and without HDM extract (10µg/ml) in vitro stimulation for 5 days are depicted. Samples were subsequently applied in keratinocyte cultures, which were finally subjected to generate representative transcriptome datasets.


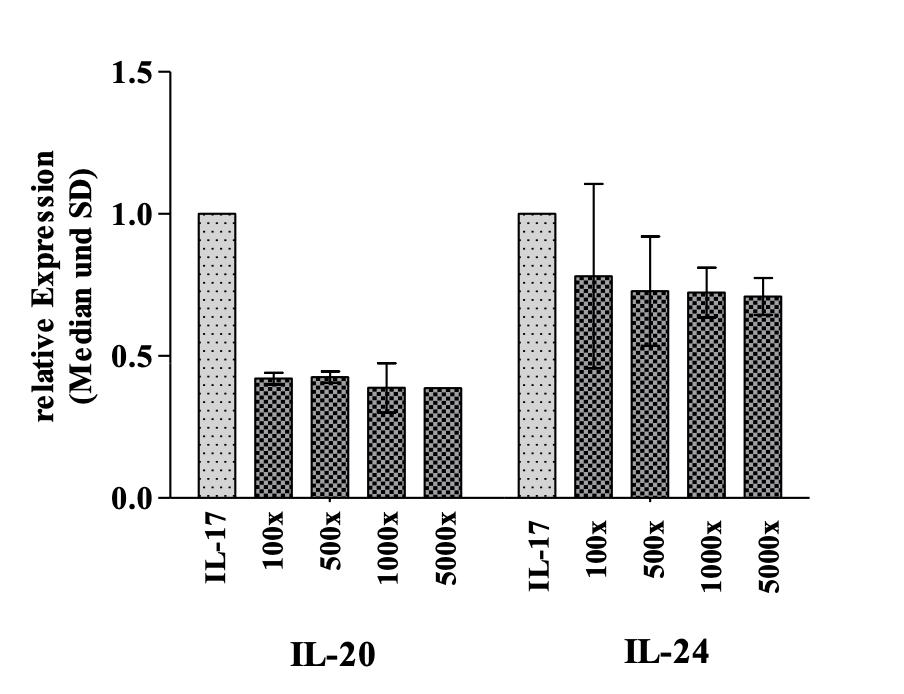


**Supplemental Figure 3. Titration of secukinumab.** Primary human keratinocytes were stimulated with recombinant IL-17 to induce the expression of the cytokines IL-20 and IL-24, as determined by realtime RT PCR. Secukinumab was added 1h before stimulation into the culture medium (dark bars). The concentration of the antibody was 100x, 500x, 1000x, or 5000x higher than the concentration of the added IL-17 (in g), respectively.


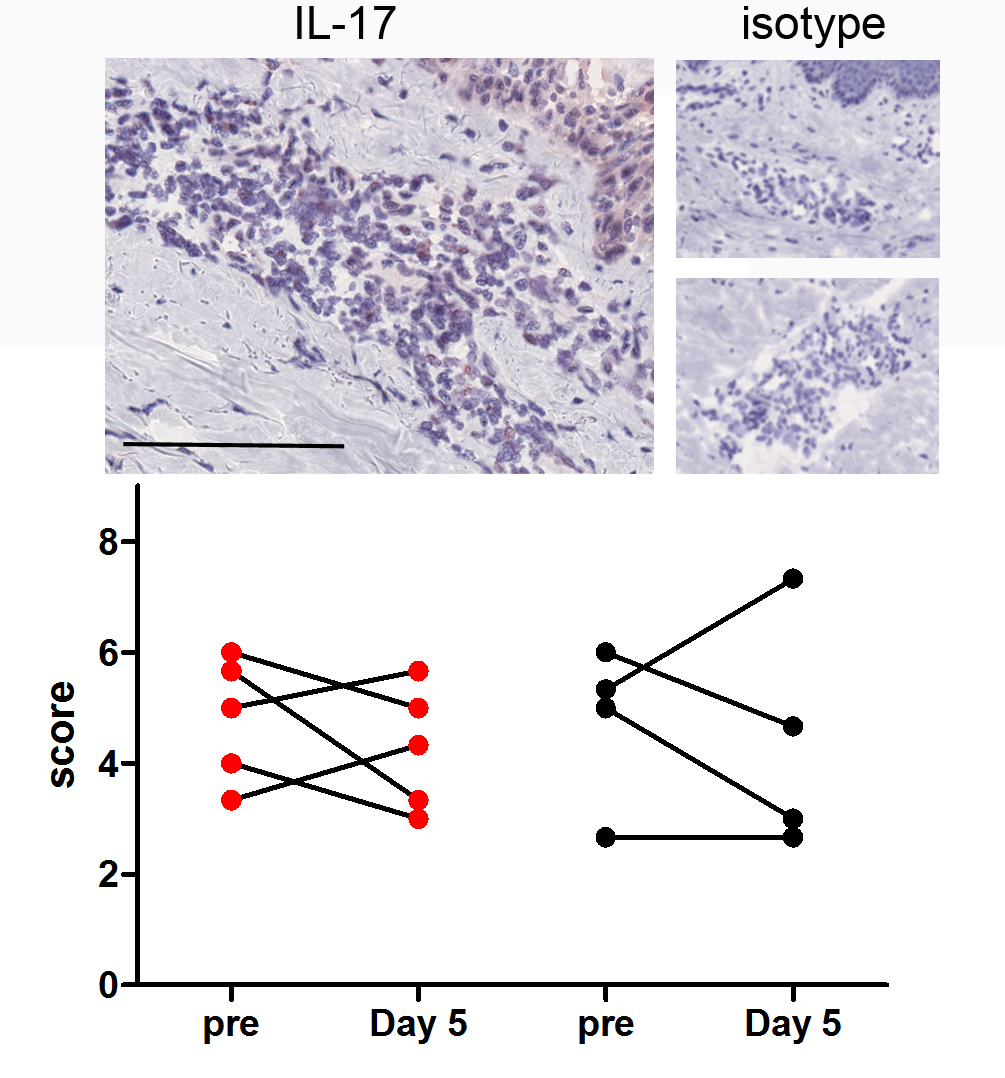


**Supplemental Figure 4.** Expression of IL-17 in lesional AD skin assessed by immunohistochemistry. IL-17 was detected by a mouse-anti-human IL-17 IgG1 (clone eBio64DEC17, Invitrogen, Carlsbad, CA, USA), followed by anti-mouse horseradish peroxidase (Dianova, Hamburg, Germany). Development was performed applying the species-specific Envision Kit (Agilent, Santa Clara, CA, USA).
